# Enhancing Oral Vaccine Potency by Targeting Intestinal M Cells

**DOI:** 10.1371/journal.ppat.1001147

**Published:** 2010-11-11

**Authors:** Ali Azizi, Ashok Kumar, Francisco Diaz-Mitoma, Jiri Mestecky

**Affiliations:** 1 Infectious Disease and Vaccine Research Center, Children's Hospital of Eastern Ontario Research Institute, Ottawa, Ontario, Canada; 2 Department of Pathology and Laboratory Medicine, University of Ottawa, Ottawa, Ontario, Canada; 3 Department of Microbiology, University of Alabama at Birmingham, Birmingham, Alabama, United States of America; 4 Institute of Immunology and Microbiology, First Faculty of Medicine, Charles University, Prague, Czech Republic; 5 Institute of Microbiology, Department of Immunology, Academy of Sciences of the Czech Republic, Prague, Czech Republic; University of California San Diego, United States of America

## Abstract

The immune system in the gastrointestinal tract plays a crucial role in the control of infection, as it constitutes the first line of defense against mucosal pathogens. The attractive features of oral immunization have led to the exploration of a variety of oral delivery systems. However, none of these oral delivery systems have been applied to existing commercial vaccines. To overcome this, a new generation of oral vaccine delivery systems that target antigens to gut-associated lymphoid tissue is required. One promising approach is to exploit the potential of microfold (M) cells by mimicking the entry of pathogens into these cells. Targeting specific receptors on the apical surface of M cells might enhance the entry of antigens, initiating the immune response and consequently leading to protection against mucosal pathogens. In this article, we briefly review the challenges associated with current oral vaccine delivery systems and discuss strategies that might potentially target mouse and human intestinal M cells.

## Advantages and Challenges Surrounding Mucosal Vaccines

The mucosal immune system is a critical line of defense against infectious diseases, as the majority of infections are initiated at mucosal sites [1]–[3]. Therefore, the induction of specific immune responses at mucosal sites may be able to control infections at their point of entry into the body. Over the past few decades, several candidate vaccines have been designed and tested by various mucosal routes in pre-clinical or clinical trials. Although the mucosal immune system comprises several anatomically remote and functionally distinct compartments, it is firmly established that the oral ingestion or intranasal administration of antigens induces humoral and cellular responses not only at the site of antigen exposure but also in other mucosal compartments [4], [5]. This is due to the dissemination of antigen-sensitized precursor B and T lymphocytes from the inductive (e.g., intestinal Peyer's patches) to the effector sites such as the above mentioned glands. However, not all inductive sites display comparable ability to induce equal responses at all effector sites. Despite several advantages, as compared to systemic injections, the delivery of vaccines by mucosal routes, particularly through the genitals or rectum, has not been shown to be very practical in human trials [6]–[8]. In addition, it is hard to administer a mucosal vaccine through the genital tract, as the immunological features of the female reproductive tract, in particular, alter dramatically in response to hormonal fluctuations during the menstrual cycle [9]–[11]. In addition, both male and female genital tracts lack inductive mucosal sites analogous to intestinal Peyer's patches [12]. Furthermore, rectal vaccinations have been shown to induce only modest and localized immune responses, and are not very effective in larger animals and humans [13], [14]. The pitfalls in quantifying effector cells in rectal tissues, combined with the intricacies of the inoculation route, are some other major challenges associated with rectal immunization. Therefore, in order to advance a mucosal vaccine for human use, the routes of administration appear to be limited to oral and nasal administration.

Nasally delivered vaccines are easy to administer and have been shown to be more promising for inducing both mucosal as well as systemic immune responses [15]–[17]. It should be stressed that the immune system of the upper respiratory tract (nasal cavity, oropharynx, trachea, and large bronchi) and lower respiratory tract (bronchioli and alveoli) display marked differences with respect to the dominance of Ig isotypes and induction of humoral immune responses. While the induction of dominant IgA responses in the upper respiratory tract is of importance in the protection at this locale, the lower respiratory tract is the domain of antibodies, of the IgG isotype of circulatory origin. Consequently, systemic immunizations with, for example, pneumococcal polysaccharide vaccines, induce protective immune responses. A nasal spray influenza vaccine (FluMist) containing live attenuated influenza has been approved for human use since 2003 [18], [19]. In an HIV study, macaques that were intranasally vaccinated with SHIV-capturing nanospheres demonstrated elevated levels of IgA and IgG antibodies [20]. Additionally, these vaccinated macaques showed a higher frequency of CD4 +T cells and lower viral loads compared to control macaques after a SHIV challenge. However, two human clinical trials involving nasal administration of HIV-1-derived antigens were recently terminated due to safety concerns. The potential for side effects such as Bell's palsy and damage to the olfactory nerves and the nasal epithelium have been cause for concern; however, these side effects could have occurred due to the use of highly reactogenic adjuvants and not because of the route of administration [21]–[23]. The possibility of such side effects, and the reason that the gastrointestinal (GI) tract is the first line of defense against mucosal pathogens, has led many scientists to pursue oral vaccination. The advantages and disadvantages of each route of mucosal immunization are summarized in Table 1. In this article, the advantages, challenges, and pitfalls with this route of vaccination are addressed. We also briefly review current options for oral delivery systems and approaches that have been explored to improve the uptake of potential vaccines. Oral vaccines have the ability to induce both mucosal and systemic immune responses and are safer, easier to administer, and do not require sterile needles and syringes [24]–[27]. Therefore, oral vaccines could more easily meet the needs of affected people in developing countries, where access to trained medical professionals is frequently limited. Although oral vaccines have several attractive features, the limited numbers of approved oral vaccines attest to the challenges associated with mucosal vaccine design. Studies involving oral vaccine use have been limited due to several challenges, such as difficulties in the collection and processing of external secretions, a lack of standardized assays, the induction of tolerance, the stability of antigens in the harsh conditions of the GI tract, and the antigen–microbial interactions that are continuously occurring in the large intestine [28], [29]. It is for these reasons that only a limited number of oral vaccines are currently licensed, compared to many parenteral vaccines.

**Table 1:** Advantages and Disadvantages of Each Route of Mucosal Immunization Is Summarized.

Route of Immunization	Advantages	Disadvantages
Genital delivery	Specific systemic and mucosal IgG and IgA antibody responses in genital secretions	Administration of antigens via male genital tract is impractical; immunological properties of the female reproductive tract alter during the menstrual cycle
Rectal delivery	Specific antibodies and cytotoxic T lymphocyte response in mucosal secretion of small animals	Modest levels of local IgG and IgA titers in human; difficulty in quantifying effector cells in rectal tissues; difficulty in the route of inoculation
Nasal delivery	Enhances both humoral and cellular immune responses in systemic and mucosal sites; easy to administer, no needles or syringes are needed	Lack of strong adjuvants; side effects such as Bell's palsy and damage to olfactory nerves and the nasal epithelium
Inhalation delivery	Enhances both humoral and cellular immune responses in systemic and mucosal sites; administered in both dry powder or liquid formulations	A device is required; risk of exacerbation of respiratory infections; difficulty in administration to infants or congested patients; dose delivery issues
Sublingual delivery	Antigens are absorbed quickly; induction of IgG in systemic sites; no needles or syringes are needed	Dose delivery issues; difficulty in formulation of antigens; lack of strong adjuvants
Oral delivery	Enhances immune responses in systemic and mucosal sites; safe; easy to administer; no health care professional is needed; easy to scale up	Induction of tolerance in some animals; requires large dose of antigens; lack of stability of antigens against the harsh conditions of the GI

## Oral Vaccine Delivery Systems

Recombinant or attenuated strains of various bacteria such as *Salmonella*, *Escherichia coli*, *Listeria*, *Shigella*, and *Lactobacilli* have been used as a vectors to deliver antigens into the gut-associated lymphoid tissue (GALT) [30]–[35]. While some interesting results have been reported for these oral delivery systems, immune responses against the vectors eventually predominated over time [36], [37]. In addition, glycosylated antigens cannot be produced in bacteria [38]. Furthermore, over 10^14^ microorganisms of >20,000 species reside in the large intestine [43]. Such a large competing population would greatly diminish the chances of colonization and subsequent induction of a vigorous immune response through such vector microorganisms. Oral delivery of live attenuated recombinant viruses such as adenoviruses (Ad), poxviruses, influenza, herpes viruses, and polioviruses encoding specific antigens has been also tested in several oral vaccine studies. While these viral vectors showed promising results, pre-existing immunity to these viruses may prevent their ability to deliver desired antigens.

Oral delivery of DNA vaccines encoding various antigens has also been evaluated in various animal studies [1], [2], [39]–[41]. DNA vaccines contain unmethylated CpG motifs with binding activity to TLR9 receptors. This characteristic assists in activating a variety of cells including dendritic cells (DCs), macrophages, monocytes, and splenocytes [42]. The TLR9 signaling pathway leads to IL-1β and INF-γ secretion, polarizing the immune response to a Th1 type. One of the pitfalls associated with DNA vaccines is the low uptake of DNA from the intestinal tract, which consequently limits B and T cell immune responses [43].

Over the past few years, specific T and B cell epitopes have been characterized in tumor and viral antigens. Synthesis of peptide epitopes for use as a vaccine requires an understanding of T and B cell immunodominant epitopes in the protein structure, and their interaction with major histocompatibility complexes (MHCs) or human leukocyte antigen (HLA) complexes [44]–[48]. The design and development of immunodominant multivalent epitopes representing diverse HLA types is an attractive strategy against hypervariable viruses such as HIV-1 and hepatitis C virus (HCV). One of the pitfalls with this approach is that peptide vaccines are not immunogenic alone, and thus require carriers and potent adjuvants to enhance their immunogenicity. The use of lipidated peptide immunogens is one of several strategies currently being pursued for the improvement of peptide immunogenicity [49]–[51]. Previous studies have demonstrated that the presence of lipid moieties on peptides prolongs the duration of antigen presentation, enhances cytosolic uptake of peptide immunogens, activates innate immunity due to a TLR2 agonist effect, and differentiates non-activated B cells into immunoglobulin-secreting plasma cells [52]–[55]. Although no commercialized peptide vaccine is yet available, this approach has shown promising results in animal studies [56]. Oral delivery of peptide vaccines has been evaluated in pre-clinical and clinical trials. In a phase I study, 33 HIV-seronegative volunteers were primed orally three times with a V3 peptide derived from HIV-1 isolate MN, followed by a systemic boosting [57]. While no broad humoral or cellular immune responses were detected, the results could prove helpful in the further development of orally administered peptide vaccines.

Plant-based oral vaccines are another delivery system that has been tested in recent years [58]–[61]. Seed crops such as rice, maize, and soybean appear to be suitable expression and delivery systems that offer several advantages, such as resistance to intestinal enzymes, rapid scale-up of exogenous antigens, low-cost production, and a decreased risk of contamination by human pathogens [62], [63]. In a mouse study, MucoRice-expressed cholera toxin subunit B (CTB) was administered orally to animals, and specific immune responses and neutralizing activity in both systemic and mucosal compartments were detected [64]. Interestingly, immunized animals with MucoRice-CTB demonstrated protection from an oral challenge with cholera toxin compared to control animals. In a similar study conducted in a non-human primate model, cynomolgus macaques received orally administered MucoRice-CTB. Animals were found to have CT-specific, neutralizing antibodies, and high levels of systemic IgG and intestinal IgA antibodies [65].

Over the past few years, several oral vaccine delivery vehicles such as liposomes, dendrimers, multiple emulsions, immune stimulating complexes (ISCOMs), biodegradable polymers such as poly (lactide-co-glycolide acid), and dendrimers have also been identified [66]–[70]. Antigens, adjuvants, and targeting molecules could be incorporated individually or in combination into these microparticles. These vehicles may thus act as immunostimulants while preventing the degradation of immunogens by enzymes in the GI tract. These particulate formulations might also interact with microfold (M) cells and release immunogens slowly, consequently promoting phagocytosis. Some microparticle studies have shown that the addition of polymers such as chitosan might increase the interaction of antigens with the intestinal mucosal surface [66], [67]. The efficacy of these microparticles has been tested in several animal studies and in a limited number of clinical trials. In a human trial, five volunteers were orally immunized with a surface *Enterotoxigenic Escherichia coli* (ETEC) polymeric protein (CS6) associated with a biodegradable polymer, poly-lactide-co-glycolide (PLG) [68]. Oral administration of these microparticles was safe, and four out of five volunteers showed IgA responses and a 3.5-fold increase in the levels of serum IgG antibody responses.

In a study by Frey et al. [69], oral administration of CTB was tested as a model for enhancing antigen uptake by intestinal epithelial cells. CTB was chosen as it promotes immune responses when co-administered orally, and its receptor (ganglioside GM1) is present on all intestinal epithelial cell surfaces. In vivo results in rabbits showed that soluble CTB-FITC (diameter of 6.4 nm) was able to bind to apical membranes of both enterocytes and M cells. Whereas CTB coupled to colloidal gold (diameter of 28.8 nm) bound only to M cells and not enterocytes, CTB-coated microparticles (diameter of 1.13 µm) failed to bind to either rabbit enterocytes or rabbit M cells. In a study by Mann et al. [70], two different sizes of a liposome-entrapped influenza antigen were delivered orally in a mouse model. The group of mice that was orally immunized with larger liposomes (60–350 nm and 400–2,500 nm) showed a greater Th1 bias, serum IgG2a production, and antigen-induced splenocyte IFN-γ production, compared to mice having received liposomes 10–100 nm in size. While this study also showed that microparticle size is an important factor associated with particle uptake, the size of the microparticles was quite different from a previous study.

However, sizing is not the only issue with these microparticles. A variety of additional parameters, including the ratio and quantity of chemical components, the amount of encapsulated antigen, hydrophobicity, the ionic surface charge, the type of associated adjuvants, and the dose of administration are also crucial, and should be optimized. In this context, the association of M cell–targeting ligands on the surface of the delivery vehicles might also enhance the binding specificity to intestinal Peyer's patches. In the next section, we briefly describe the M cell surface markers that could be considered in a strategy to enhance capture and uptake of orally administered vaccines.

## Targeting the Apical Surface of M Cells

**Figure 1:**
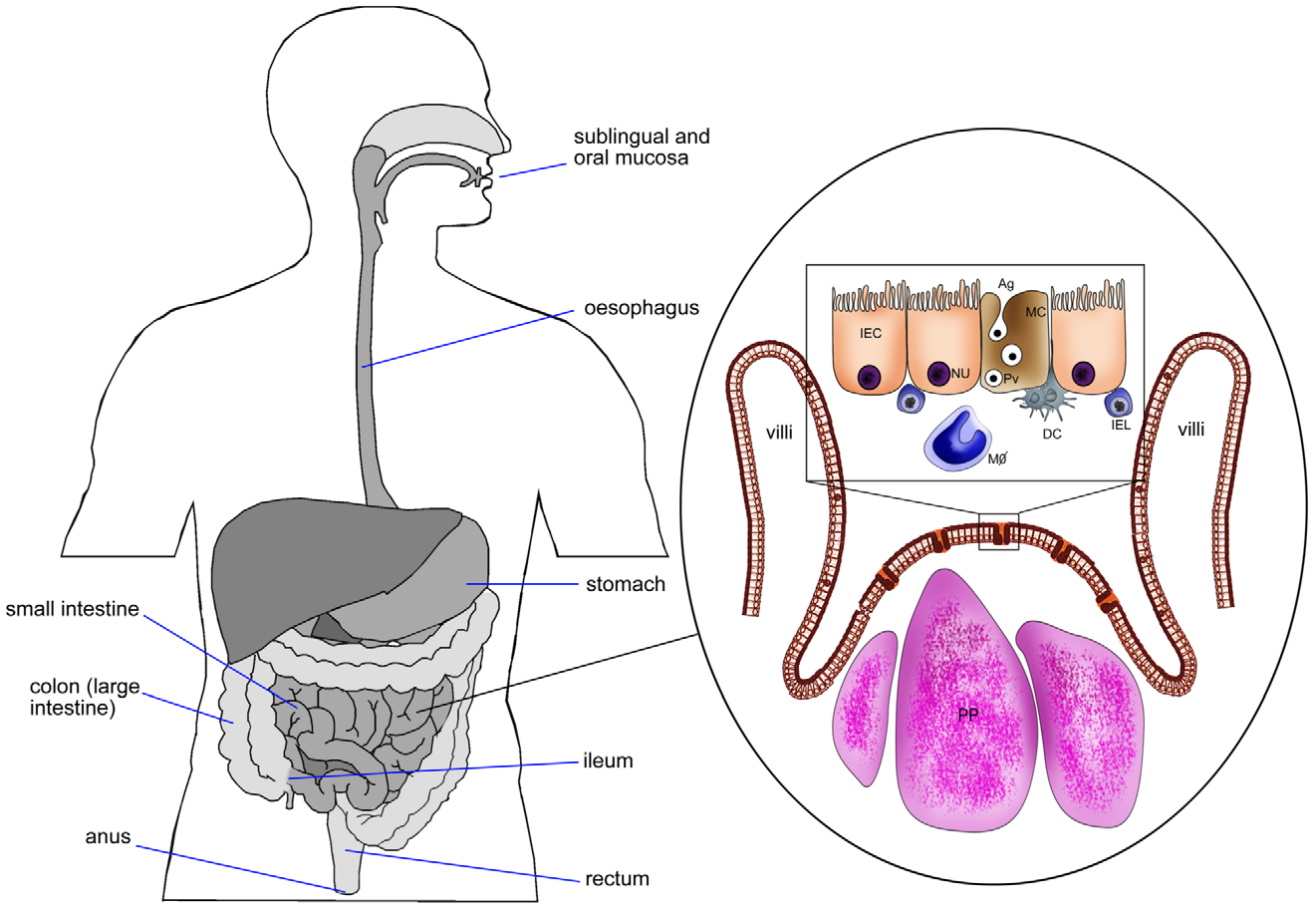
Schematic diagram of intestinal epithelium showing M cells, Peyer's patches, intestinal epithelial cells, and pathway of Ag transport. DC, dendritic cells; IEC, intestinal epithelial cell (NU, nucleus); MC, M cell; IEL, intra epithelial lymphocytes; PP, Peyer's patches; MΦ, macrophages; Pv, particulate Ag in pinocytic vesicle of M cell.

M cells are specialized epithelial cells that predominantly reside in the follicle-associated epithelium (FAE) overlying Peyer's patches. M cells also reside in other sections of the intestinal tract such as the colon and rectum [71]–[74]. M cells are identifiable by their flattened apical surfaces, fewer numbers of cytoplasmic lysosomes, greater numbers of mitochondria, and the absence of glycocalyx covering their surfaces. It also appears that mouse M cells express particular surface markers, compared to enterocytes, such as β1 integrin or α-L-fucose-specific (L-fucose) lectin [75]. In contrast to enterocytes, M cells take up antigens or microorganisms from the intestinal lumen (Figure 1) by phago-, endo-, or pinocytosis and transcytosis, and deliver them to the underlying immune system of the mucosae. This phenomenon also occurs by other mechanisms, for instance in intestinal DCs; however, this will not be discussed here. M cells are not limited to the GALTs, and are also present in other mucosal tissues such as nasopharyngeal-associated lymphoid tissue (NALT) and bronchus-associated lymphoid tissues (BALT) and tonsils [76], [77]. It has been shown that M cells in NALT are a major site of virus entry as well as vaccine delivery; however, limited studies have been reported with regards to the roles of NALT and BALT in the uptake and transport of vaccine-delivered antigens.

The ability of M cells in Peyer's patches to take up and transcytose diverse numbers of microorganisms to antigen-presenting cells (APCs) have made M cells an ideal target for vaccine delivery to the mucosal immune system [78]–[80]. It is estimated that only 1 out of 10 million epithelial cells in the intestinal tract is an M cell (approximately 5% in humans and 10% in mice) [81]. Due to these low numbers of M cells, several approaches have been attempted to enhance M cell targeting. It has been indicated that M cell numbers in Peyer's patches are increased after exposure to *Streptococcus pneumonia* R36a [82]. However, these increased numbers of M cells may uptake all antigens in the intestinal epithelium and not just the antigens of interest, consequently increasing the probability of inducing food allergies and inflammatory diseases. Therefore, it might be more reasonable to target the existing M cells in Peyer's patches than to try to amplify their numbers.

Targeting specific receptors on the apical surface of M cells may have the ability to specifically increase the uptake and presentation of antigens, consequently initiating the immune response and inducing protection against infectious challenge. To date, only limited numbers of M cell receptors and their ligands have been identified, and most of these receptors are not only expressed in M cells but in neighboring enterocytes as well. Some important pathogen recognition receptors (PRRs), such as toll-like receptor-4 (TLR-4), platelet-activating factor receptor (PAFR), and α5β1 integrin, are expressed on the surface of human and mouse M cells [83]–[85]. These innate immune system molecules interact with pathogen-associated molecular patterns (PAMPs) such as lipopolysaccharide, lipotechoic acid, peptidoglycan, and bacterial flagellin. This interaction is crucial for the translocation of bacteria across the lumen. Consequently, targeting PRRs might be a suitable strategy for enhancing the uptake of orally administered vaccines by M cells. This interaction activates several signaling pathways that may play important roles in M cell functions. For instance, M cells take up many enteropathogenic microorganisms, such as *Yersinia* spp., via the α5β1 integrin, and inhibition of this adhesion molecule significantly inhibits transcytosis of M cells [86]–[88]. While PRRs are also expressed on neighboring enterocytes (a challenge in targeting only M cells), the expression patterns of these receptors are varied. For instance, α5β1 integrin is dispersed on the lateral and basolateral surfaces of enterocytes, while in M cells, α5β1 is distributed only on the apical surface.

Lectin-binding studies in experimental animals have shown that M cells express on their surface a particular glycosylation pattern [89], [90]. Several studies showed that *Ulex europaeus* agglutinin-1 (UEA-1), a lectin specific for α-l-fucose residues, selectively binds to M cells in murine Peyer's patches [91]–[94]. In a study by Manocha et al. [94], the UEA-1 coated on the surface of microparticles encoding HIV genes had the capability to bind to the apical surface of M cells. In another study, by Chionh et al. [95], oral vaccination in a mouse model with killed whole *Helicobacter pylori* and UEA-1 or *Campylobacter jejuni* and UEA-1 induced protective responses against live challenge. However, M cell glycosylation patterns are not common to all species, and it remains to be seen whether it can be used to effectively target human M cells [96]. Human M cells have proven to be largely anonymous, as it has been difficult to isolate enough of such cells for further characterization and functional evaluation. Therefore, the specific receptor requirements for human M cells and how to specifically target these receptors remains a challenge. In recent years, a few in vitro human M cell models have been established [97], [98]. One of the most common M cell–like models is comprised of co-cultures of human colon carcinoma cells (Caco-2) along with human lymphoblastoid B cell lines (Raji B cells) [99], [100]. This in vitro model has been used to study the morphology and expression of M cell surface markers and antigen absorption, and to screen oral drug/vaccine delivery systems, as it closely imitates human M cells. While these M cell–like models have been used to attempt to further understand human M cells, one of the concerns of this model pertains to its over-simplification of in vivo events, as well as the lack of signaling factors from other immune cells such as T cells that are required for the formation and optimal function of M cells.

Microarray and three-dimensional imaging of specific molecules associated with M cells has revealed that a surface marker called glycoprotein 2 (GP2) is expressed on both human and mouse M cells [101], [102]. It appears that GP2 plays an important role in molecular mechanisms responsible for antigen uptake by M cells. GP2 serves as a transcytotic apical receptor on the surface of M cells that specifically binds to type I pili on bacterial outer membranes (FimH) [101]. Elimination of GP2 reduced the entry and uptake of bacteria into Peyer's patches and decreased T cell proliferative and antibody responses. Altogether, these results suggest that the GP2 protein might be a promising vaccine target for immunizing against infectious diseases. Several studies have shown that FimH adhesion–based vaccines are able to prevent infection by impeding colonization, enhancing humoral immune responses, and blocking bacterial attachment [103]–[105]. It would be exciting to determine if FimH could direct other antigens to M cells as well.

In an interesting study by Giannasca et al. [106], Peyer's patches were biopsied from volunteers with blood groups type O (two individuals) and type A (one individual). The binding and cellular localizations of 31 lectins and ten anticarbohydrate monoclonal antibodies from biopsy samples were performed by histochemistry and compared to the nearby enterocytes on Peyer's patches. Lectin and antibody results revealed a higher expression of carbohydrates on enterocytes than M cells. Some lectins and antibodies such as OPA and anti-Lewis A also reacted with both M cells and enterocytes. Interestingly, only one (anti-sialyl Lewis A) out of the 41 tested lectins or antibodies largely reacted with human M cells (∼80%) and bound only weakly to the FAE enterocytes (∼20%). While a larger number of human tissue specimens are required to confirm this oligosaccharide repertoire, an anti-sialyl Lewis A–mediated vaccine delivery system might be appropriate approach to enable M cell–targeted mucosal vaccines in humans.

In a study by Misumi et al. [107], the capability of tetragalloyl-D-lysine dendrimer (TGDK) to target M cells was examined in an in vitro human M–like cell culture and a rhesus macaque animal model. The results indicated that TGDK specifically bound to a human intestinal M–cell like model under in vitro conditions and was delivered from the M cell surface to the basolateral area. To examine the in vivo effect of TGDK on M cell targeting, rhesus macaques were orally administered with enteric coated capsules containing TGDK-conjugated multiantigens at weeks 0, 2, and 6. ELISA from feces samples of immunized macaques indicated a high level of IgA antibody responses. Conversely, the control macaques did not induce specific IgA in fecal samples. Furthermore, the immunized macaques with TGDK-conjugated multiantigens also showed neutralizing activity against SIV infection. These results concluded that TGDK transports from the lumen into intestinal M cells, and can consequently be considered for use in mucosal vaccine delivery in humans and non-human primates.

## Mucosal Immune Responses and Mucosal Tolerance

Repeated oral administration of large doses of antigen in animal models result in decreased or abrogated T cell–mediated responses to a subsequent systemic immunization with the same antigen [108]. This phenomenon prompts a question concerning the possible induction of mucosal tolerance by mucosally delivered vaccines. Importantly, for vaccine efficacy the dominant target of oral tolerance is the T and not the B cell compartment. As a matter of fact, initial mucosal administration of antigens by the oral or nasal routes primes for B cell responses in parallel with diminished T cell responses in humans as well as in animals [109]. Thus, vaccines whose protective effect is dependent on the induction of antibodies (which is the target of all currently used vaccines in humans) are not likely to diminish their efficacy by mucosal administration of antigens. Furthermore, pre-existing immune responses induced by systemic immunization cannot be attenuated or suppressed by subsequent mucosal administration of the same antigen [109]. However, initial mucosal immunization of immunologically naïve subjects (e.g., with HIV-1 vaccines) might have the undesirable effect of diminishing cell-mediated responses, including cytotoxic T cell–dependent immunity. Thus, the temporal sequence of immunization with initial systemic priming and mucosal boosting as well as the use of certain adjuvants is likely to prevent the induction of mucosal tolerance.

## Concluding Remarks

Over the past few decades, oral immunization has been extensively studied due to its many attractive features. The immunological potential, absorption, or limitation in the uptake of antigens, as well as the characteristic distribution of functional cell types in the GI tract, have made it a vital target in the development of oral vaccines. The phenomenon of tolerance is a crucial challenge to overcome in the development of effective oral vaccines. Experimental animal studies have indicated that oral administration of antigens targets the systemic T cell compartment, diminishes cell-mediated immune responses, and induces tolerance. This phenomenon might lead to the induction of cytokines such as TGF-β and IL-10, and consequently enhance antigen-specific antibody responses such as IgA and IgG. While the humoral immune response is critical in the control of some mucosal pathogens, its effect might be questionable on other mucosal pathogens such as HIV and HCV where cell-mediated immune responses may play a larger role. Opponents to this tolerance hypothesis, including the authors of this article, believe that tolerance is not an issue in humans, as it occurs through a completely different mechanism. Furthermore, some clinical studies have showed that a combination of oral priming and systemic boosting might activate both humoral and cellular arms of the immune system. On the other hand, we think that the absence of a potent oral vaccine might be due to other challenges, including antigen degradation by proteolytic enzymes, the low dose of antigen absorbed, a lack of potent mucosal adjuvants, and not actively directing antigens to M cells. To overcome these issues, further work regarding oral vehicle delivery systems that protect antigens and specifically target M cells is required. Targeting M cells by mimicking the entry of mucosal pathogens such as *E. coli*, *Salmonella*, and *Yersinia* may reflect the in vivo binding specificity required by orally administered antigens. Regarding this aspect, a number of studies showed that these pathogens bind to specific lectins expressed on the apical surface of M cells. The binding of orally administered vaccines to M cell lectins was further studied in murine models and indicated that α-L-fucose-lectin (UEA-1) is able to bind specifically to M cells and, to a lesser degree, enterocytes. However, the characterization of murine M cells by this lectin-binding pattern did not reflect the glycosylation patterns present on human M cells. Unfortunately, human M cell features, function, and differentiation from neighboring enterocytes are not well understood.

Based on previous studies, by using tetragalloyl-D-lysine dendrimers, a monoclonal antibody targeting GP2, or using a monoclonal antibody targeting sialyl Lewis A, it might be possible to more specifically direct oral delivery systems to human M cells. However, as these molecules are also expressed on neighboring enterocytes (albeit at lower levels), it will likely be difficult to devise an ideal oral delivery system for targeting human M cells. The understanding of human M cell function, identification of more specific apical surface molecules, and the improvement of intestinal M cell–like models are crucial for the design and further development of M cell–targeted vaccines.
